# Effects of dietary N-carbamylglutamate in supplemental feed on lactation performance of grazing yak cows

**DOI:** 10.5713/ab.260356

**Published:** 2026-09-01

**Authors:** Yuhong Bao, Zhenyu Zhu, Jia Zhou, Ke Feng, Ali Mujtaba Shah, Lizhuang Hao, Yangci Liao

**Affiliations:** 1 Institute of Grassland Research of Xizang Academy of Agriculture and Animal Husbandry Sciences, Lhasa, China; 2 College of Animal Science and Veterinary Medicine, Qinghai University, Xining, China; 3 Chongqing Academy of Animal Sciences, Chongqing, China; 4 Center of Lhasa Animal Disease Prevention and Control, Lhasa, China; 5 State Key Laboratory of Swine and Poultry Breeding Industry, College of Animal Science, South China Agricultural University, Guangzhou, China

**Keywords:** Cold-season Nutritional Intervention, Late-lactation Ruminants, Milk Fat Synthesis, Milk Yield, N-carbamylglutamate, Serum Amino Acids

## Abstract

**Objective:**

Yak milk serves as crucial source of dairy products for pastoralists on the Qinghai-Tibetan Plateau. This study investigated the effects of dietary N-carbamylglutamate (NCG) in supplemental feed on lactation performance, milk composition, serum biochemical parameters, and free amino acid profiles in grazing yaks.

**Methods:**

Twenty-seven late-lactation Niangya yaks (2.33±0.68 parity; 164.85±9.58 days in milk; 239.50±36.37 kg BW) were randomly assigned to three groups (n = 9). Yaks grazed spontaneously during the day and were penned individually at night to receive a 1% BW supplement of Control, NCG1 (7.5 g/d), or NCG2 (15 g/d). Following a seven-day adaptation, the experimental trial lasted 60 days.

**Results:**

The study showed that NCG supplementation significantly increased (p<0.05) milk yield compared with the control group. On day 60, the NCG1 group had a significantly higher (p<0.05) milk fat percentage than those of both the control and NCG2 groups. On day 30, the NCG1 group exhibited significantly (p<0.05) elevated serum concentrations of arginine, alanine, and valine relative to the control group, along with higher (p<0.05) valine, isoleucine, and leucine levels compared to the NCG2 group. Both the NCG1 and NCG2 groups had increased (p<0.05) levels of serum alanine compared with the control group.

**Conclusion:**

Collectively, milk yield increased linearly with NCG dosage and peaked at 15 g/d, whereas milk fat percentage exhibited a quadratic response, peaking at 7.5 g/d. This suggests that moderate NCG supplementation (7.5 g/d) optimally promotes milk fat synthesis, while a higher dose (15 g/d) further enhances milk volume without conferring additional benefits on fat content. The dose-specific responses point to a potential dissociation between pathways regulating mammary lipogenesis and amino acid-driven lactogenic signaling; nevertheless, these mechanistic hypotheses require a direct experimental confirmation.

## INTRODUCTION

The yak (*Bos grunniens*), as the primary livestock species on the Qinghai-Tibet Plateau (QTP), inhabits regions with average altitudes exceeding 3,000 m characterized by hypoxic conditions and extreme cold environments [1]. Yaks serve as multifunctional livelihood assets for herders on the QTP, delivering critical provisioning services that encompass nutritional resources (meat and milk), household energy (dung fuel) and construction materials (hides) [2], thereby forming an irreplaceable foundation for high-altitude subsistence strategies. The lactation yield of yaks typically ranges from 150 to 500 kg per lactation period [3], with variations primarily determined by genetic factors (including breed, age, and parity) and physiological status [4]. Yak milk demonstrates superior nutritional properties compared to Holstein dairy, with significantly higher protein and fat contents, enhanced bioactive compounds, and unique microbial profiles [5]. In the resource-scarce alpine pastoral systems of the QTP, traditional yak dairy products like butter and cheese serve as essential dietary staples and crucial sources of vitamins and micronutrients for Tibetan herders [3,4,6]. Despite the lack of standardized global production data (current estimates range from 0.7 to 40 million tons annually), yak milk plays an indispensable role as both the primary nutritional source and principal economic foundation for pastoral communities inhabiting high-altitude ecosystems [4,7]. Consequently, enhancing yak milk production efficiency has emerged as a critical research priority with significant implications for ensuring food security, preserving nomadic cultural traditions, and maintaining ecological balance in these fragile alpine ecosystems.

On the QTP, the harsh cold season extends for nearly 8 months (October to following May, with an average temperature below −5°C) [8], during which both the biomass and nutritional quality of natural pasture experience dramatic declines, creating severe nutritional deficits for grazing yaks [9]. Yaks are typical seasonal breeders, with most estrus occurring in July and August, and calving taking place the following April to May (gestation period: 250–260 days) [10]. Yaks experience progressive declines in milk yield and body condition during late lactation in the prolonged cold season, where nutritional demands for lactation critically mismatch with seasonal forage scarcity under extreme high-altitude conditions [11,12]. Previous studies have confirmed that supplementary feeding during cold seasons significantly improves production performance and body condition in yaks [13–15], potentially benefiting milk production. N-carbamylglutamate (NCG), a functional analog of N-acetylglutamate, is a known activator of endogenous arginine synthesis in mammals [16], has been demonstrated to significantly enhance milk production and quality parameters (including fat and protein contents) in Holstein dairy cattle confined feeding systems [17,18]. However, research on the effects of NCG in grazing ruminants, particularly those in extreme environments like the QTP, remains scarce. Furthermore, it is unknown whether NCG can synergize with conventional supplementation strategies to improve lactation performance in yaks during the cold season, when nutrient demands for milk production are challenged by limited forage quality and availability. We therefore hypothesized that dietary NCG supplementation, when added to a standard supplemental feed, would enhance milk yield and modify milk composition in grazing yaks by improving the profile of serum amino acids critical for lactation.

Thus, this study aimed to investigate the effects of NCG supplementation on both lactation performance and milk quality in grazing yaks, while evaluating their physiological and metabolic status through serum biochemical parameters and amino acid profiles.

## MATERIALS AND METHODS

### Experimental design and animal management

This study was conducted from November 2023 to February 2024 in Nagqu, Tibet (92.12°–92.34°E, 31.64°–31.87°N; altitude: over 4,500 m), with an average daily temperature of −3.2 to 3.4°C during the trial period. Twenty-seven late-lactation Niangya yaks (parity: 2.33±0.68; days in milk: 164.85±9.58 d; body weight: 239.50±36.37 kg) were randomly allocated into three treatment groups (n = 9): (1) Control (0 g NCG/d); (2) T1 (7.5 g NCG/d); (3) T2 (15 g NCG/d). The sample size was determined a priori using power analysis (G*Power 3.1) based on the following assumptions: milk yield as the primary outcome, an expected difference of 100 g/d between treatment groups (derived from previous NCG studies in dairy cattle reporting 5.6%–8.3% increases in milk yield [19,20]), a standard deviation of 85 g/d (estimated from preliminary data in our research herd and consistent with variability reported in similar studies [20], a type I error rate (α) of 0.05, and a desired statistical power (1-β) of 0.80. Under these assumptions, a minimum of 8 animals per group was required; we included 9 animals per group to account for potential attrition during the 60-day trial. The NCG supplementation levels (7.5 and 15 g/d) were designed based on previous studies in yaks (6 g/d) [21], and dairy cattle (20 g/d) [22]. Randomization was performed using a computer-generated random number sequence in Excel 2021 (Microsoft). Yaks were first stratified by parity to ensure balanced distribution across treatment groups, and then randomly assigned within each stratum to one of three treatment groups using a simple random sampling procedure. This stratification approach minimized potential confounding effects of parity on lactation performance outcomes. Yaks were housed in individual pens during the night to allow for individual feeding and milk collection. During the daytime (09:00 to 17:00), yaks were allowed to graze freely on native alpine meadows. Yaks were selected according to a standardized set of criteria, namely: days in milk ranging from 150 to 180 d, parity between 1 and 4, and body weight between 200 and 280 kg, the latter adopted to minimize inter-individual variation in feed intake capacity. All selected animals were clinically healthy, as confirmed by physical examination and a negative California Mastitis Test, and shared a consistent management history within the same grazing area prior to the experiment. Yaks with a history of metabolic or reproductive disorders were excluded from the study. The supplementary feed was formulated with corn, soybean meal, and oat hay as its base components, referencing the standard NY/T815-2004 (Table 1) [23]. Following a 7-day adaptation period, the formal experiment lasted for 60 days.

### Body weight measurement

All experimental yaks were weighed on a platform scale before morning grazing at the day 0, 30, and 60 of the trial. Individual body weights were recorded, and average daily gain (ADG) was calculated for the 0–30 d and 30–60 d intervals using the respective weight changes divided by the number of days in each period.

### Sample collection

During the experimental period, yaks were milked every morning before feeding. Simultaneously, milk yield was recorded by weight, and milk samples were collected and immediately refrigerated for subsequent analysis. On days 30 and 60 of the experiment, approximately 5 mL of jugular venous blood was collected from all yaks using vacuum tubes. After allowing the samples to clot at room temperature for 1 h, these samples were centrifuged at 800 ×g for 10 min to separate the serum. The obtained serum was immediately aliquoted and stored at −80°C for subsequent biochemical analyses.

### Sample analysis

#### Milk composition

The concentrations of fat, protein, lactose, acetone and milk urea nitrogen in yak milk were analyzed using a CombiFoss FT+ integrated milk analyzer (Foss Analytical) following manufacturer’s recommended calibration procedures. The instrument was calibrated prior to analysis using certified reference milk samples following the manufacturer’s specified protocols.

#### Serum biochemical parameters

Serum biochemical profiles were assessed using a Hitachi 7020 automated clinical analyzer (Hitachi), measuring the following parameters: total protein (TP), albumin (ALB), glucose, blood urea nitrogen (BUN), total cholesterol (TC), and non-esterified fatty acids (NEFA). Globulin level was derived mathematically by subtracting ALB from TP values.

#### Serum free amino acids

The serum free amino acid composition was determined using an Agilent 1260 high performance liquid chromatography (HPLC) system (Agilent Technologies) with a C_18_ SHISEIDO column (4.6 mm×250 mm, 5 μm). Sample preparation involved deproteinizing 1 mL serum with 2 mL of 10% sulfosalicylic acid, followed by vortex mixing, centrifugation (8,900×g, 10 min), and filtration (0.22 μm). Chromatographic separation was achieved using a binary mobile phase system: (A) 0.1 mol/L sodium acetate buffer (pH 6.5)-acetonitrile (97:3, v/v) and (B) acetonitrile-water (80:20, v/v), delivered at 1.0 mL/min with column temperature maintained at 40°C. The elution profile was optimized as: initial 100% A, linearly decreased to 85% A over 14 min, further reduced to 66% A by 29 min, then rapidly shifted to 100% B at 30 min and held for 7 min, before returning to initial conditions at 38 min with a 7-min re-equilibration. Detection was performed at 254 nm with 10 μL injection volume. Identification and quantification were based on retention time matching with standards and external calibration curves, respectively. All amino acid standards (≥99.5% purity) were purchased from ANPEL Laboratory Technologies.

### Statistical analysis

The experimental data were subjected to statistical analysis using SPSS 26.0 (IBM). All results were expressed as least squares means±standard error of the mean (SEM). For outcomes measured at multiple time points (milk yield, milk composition, serum biochemical parameters, and serum free amino acids), data were analyzed using linear mixed-effects models with repeated measures, with treatment group, time, and their interaction as fixed effects, and animal as a random effect. A compound symmetry covariance structure was specified to model the within-subject correlations. Initial body weight was included as a covariate for body weight analysis, and initial milk yield was included as a covariate for milk production analysis. Given the structured nature of the NCG treatments (0, 7.5, and 15 g/d), orthogonal polynomial contrasts were performed to evaluate linear and quadratic dose-response relationships when the overall treatment effect was significant; otherwise, contrasts are presented for descriptive purposes. Least squares means were compared using Bonferroni-adjusted pairwise comparisons, with p<0.05 considered statistically significant.

## RESULTS

### Body weight change

As presented in Tables 2, 3, all three yak groups exhibited negative weight growth throughout the experimental period, and no statistically significant differences (p>0.05) were detected among the groups. Mixed-model analysis revealed no significant treatment effects or treatment×time interactions for body weight or ADG (p>0.05).

### Lactation performance

As shown in Figure 1, with the experiment progressed, the milk yield of all trial yaks gradually increased, rising from the initial 343 g/day at the start of the experiment to 707.64 g/day (Control group), 626.76 g/day (NCG1 group), and 776.46 g/day (NCG2 group). As shown in Table 3, compared with the control group, both experimental groups (NCG1 and NCG2) exhibited significantly higher (p<0.05) average daily milk yields during the 0–30-day and 30–60 day experimental periods. Notably, the NCG2 group demonstrated superior (p<0.05) milk production compared to NCG1 specifically during the 30–60 day. Orthogonal polynomial contrast analysis revealed significant linear dose-response relationships for milk yield during all three periods (day 0–30: p = 0.018; day 30–60: p<0.001). Mixed-model analysis revealed a significant effect of treatment (p<0.001) and time (p<0.001) on milk production, with no significant treatment×time interaction (p = 0.320).

### Milk composition

Mixed-model analysis revealed no significant treatment effects on most milk composition parameters. However, milk fat percentage was significantly influenced by time (p<0.05) (Table 4). At day 30, no significant differences in milk fat percentage were observed among the three groups (p>0.05). Orthogonal polynomial contrast analysis revealed a significant quadratic dose-response relationship for milk fat percentage at day 60 (p = 0.041). For acetone concentration at day 60, a significant quadratic trend was also detected (p = 0.039, Table 4). No significant treatment effects or treatment×time interactions were observed for milk protein, lactose, or milk urea nitrogen (p>0.05).

### Serum biochemical parameters

Mixed-model analysis showed significant time effects were detected for glucose (p<0.001) and NEFA (p = 0.004), with values increasing from day 30 to day 60 across all treatment groups (Table 5). No significant time effects were observed for TP, ALB, globulin, BUN, or TC (p>0.05). The addition of 7.5 or 15 g/head/day of NCG in the supplementary feed showed no significant effects (p>0.05) on serum biochemical parameters of grazing yak dairy cows (Table 5).

### Serum free amino acids

Mixed-model analysis revealed that compared to the control group at day 30, the NCG1 group exhibited significantly increased (p<0.05) serum concentrations of arginine, alanine, valine, and phenylalanine, whereas the NCG2 group showed elevated (p<0.05) alanine and phenylalanine levels (Table 6). Furthermore, at day 30, the NCG1 group maintained higher (p<0.05) concentrations of branched-chain amino acids (BCAA; valine, isoleucine, and leucine) compared to NCG2. However, by day 60, no significant differences (p>0.05) were observed in any amino acid levels among the three groups. Orthogonal polynomial contrast analysis at day 30 revealed significant quadratic dose-response relationships for valine (p = 0.010), leucine (p = 0.015), alanine (p = 0.018), phenylalanine (p = 0.038), and arginine (p = 0.045), with NCG1 showing the highest concentrations among the three groups. A significant linear relationship was observed for phenylalanine (p = 0.021). At day 60, significant linear trends were observed for serine (p = 0.034) and histidine (p = 0.028), while aspartate exhibited a significant quadratic pattern (p = 0.044, Table 6).

## DISCUSSION

While markedly inferior in volumetric output to conventional dairy species, yak milk remains indispensable to the socioeconomic fabric and traditional lifeways of high-altitude pastoral communities. The traditional diet of Tibetan pastoralists, while lacking in vegetables and fruits, does not lead to micronutrient deficiencies, as the nutritional profile of yak milk appears to compensate for these dietary gaps [5,24]. The unique plateau environment has endowed yak milk with scientifically proven bioactive properties including hypoxia tolerance enhancement, anti-fatigue effects, antioxidant activity, and immunomodulatory capacity, making it both an economic cornerstone for pastoral communities through dairy products and a key sustainable component of highland agricultural systems [5].

Recent work has shown that its composition undergoes characteristic changes during lactation, accompanied by dynamic shifts in serum metabolites and gut microbiota [25]. For grazing yaks under harsh cold-season conditions, body condition scores typically decline, leading to significantly reduced milk production [14,26]. In this study, yak supplementation with feed at 1% of BW failed to prevent negative weight gain, confirming the extreme nutritional stress experienced during the cold season on the QTP. Mixed-model analysis revealed no significant treatment effects or treatment×time interactions for body weight or ADG, consistent with the absence of NCG effects on these parameters. An earlier study demonstrated that although supplementing grazing lactating yaks (above 230 kg) with oat hay and highland barley straw during the cold season (from December to following April) partially alleviated weight loss, the yaks still exhibited negative weight gain [12]. In comparison to male yaks, female yaks exhibited a lower weight gain response to supplemental feeding, a trend potentially associated with gender-specific resource allocation patterns in this species [14]. Another controlled feeding trial revealed that non-lactating adult male yaks (204.90±10.65 kg) maintained stable body weight during the winter grazing period when provided with a 0.5 kg/day concentrate supplement [15]. These differences may be attributed to the substantial nutritional demands of lactation in female yaks, suggesting that supplementation regimens require adjustments based on gender and physiological status.

Despite cold-season supplementation with oat hay and barley straw failing to prevent weight loss in grazing yaks, the milking period lengthened to 142 and 138 days (29- and 25-day increased) versus 113 days in controls (without supplementation) [12]. As expected, this study revealed a progressive increase in yak milk yield with extended supplemental feeding duration, suggesting that nutritional supplementation may have improved the body condition of all experimental animals. In the present study, mixed-model analysis demonstrated a significant treatment effect and time effect on milk production, with no significant treatment×time interaction, indicating that NCG supplementation consistently enhanced milk yield across both time points. Previous studies have demonstrated that NCG supplementation significantly enhanced lactation performance and nitrogen efficiency in dairy cows and lactating goats by modulating plasma metabolites, increasing arginine and nitric oxide (NO) concentrations, and improving amino acid supply and metabolic protein utilization [19,27,28]. During heat stress exposure, NCG supplementation effectively upregulates antioxidant defenses and modulates immune responses, leading to significant stress mitigation and consequent 5.3% improvement in milk production [29]. The yaks in the current study, subjected to starvation and cold stress conditions, likely experienced concurrent stress challenges, wherein dietary NCG addition demonstrated beneficial effects. The differential responses between NCG1 (7.5 g/d) and NCG2 (15 g/d) groups present a dose-response relationship. Moderate-dose NCG supplementation (20–25 g/day for dairy cattle, 2 g/day for goats) was more effective in enhancing milk yield under both heat-stress and thermoneutral conditions [19,27,29]. Interestingly, the beneficial effects of some dietary additives in dairy cows, such as blooming Ulva sp., have been reported to be transient and do not persist throughout the entire experimental period [30]. In contrast, the consistent milk yield enhancement observed across both time points in the current study suggests that NCG’s lactational benefits may be sustained under cold-season grazing conditions. The superior milk fat percentage in NCG1 compared to both control and NCG2 groups by day 60 indicates that moderate NCG supplementation appears to be more effective than the higher dose for optimizing yak milk composition under the conditions of this study. Mixed-model analysis further confirmed a significant treatment×time interaction for milk fat percentage (p = 0.039), with the quadratic dose-response relationship at day 60 (p = 0.041) is consistent with, but does not prove, the hypothesis that moderate NCG may optimally activate the mTOR/SREBP1 pathway to stimulate mammary lipogenesis, whereas higher doses may shift BCAA utilization toward energy production rather than fat synthesis [31,32]. Direct measurements of mammary tissue mTOR signaling and lipogenic gene expression would be required to confirm this speculation. Therefore, the results of this study demonstrate that incorporating NCG into supplemental feed for yaks during the cold season on the QTP can effectively enhance milk production. The significant linear and quadratic dose-response relationships for milk yield suggest that while NCG promotes milk production in a dose-dependent manner, the diminished incremental benefit at 15 g/d may reflect saturation of amino acid transporter systems or feedback inhibition of mTOR signaling at higher arginine concentrations [32]. Furthermore, the absence of significant treatment×time interactions for milk production suggests that the lactational response to NCG was stable over the 60-day experimental period.

Changes in the serum amino acid profile following NCG supplementation offer valuable insights into the underlying metabolic mechanisms. In the current study, NCG supplementation elevated serum arginine, alanine and phenylalanine compared to the control group. Mixed-model analysis revealed significant quadratic dose-response relationships for arginine (p = 0.045), alanine (p = 0.018), valine (p = 0.010), leucine (p = 0.015), and phenylalanine (p = 0.038) at day 30, with NCG1 (7.5 g/d) showing the highest circulating concentrations among the three groups. At day 60, however, no significant differences were observed among groups for any amino acid, suggesting that the NCG-induced metabolic changes were transient. These metabolic modifications are consistent with the characteristic functions of NCG in urea cycle regulation and arginine biosynthesis [28,33], demonstrating comparable efficacy for yaks grazing on high-altitude pastures during cold seasons. The concurrent increases in alanine and BCAA in the NCG1 group may be mechanistically linked to NCG’s primary action on the urea cycle. Specifically, NCG is known to activate carbamoyl phosphate synthetase I (CPS-I), the rate-limiting enzyme of the urea cycle, thereby enhancing endogenous arginine synthesis [34]. Although we did not directly measure CPS-I enzyme activity in liver tissue—which would require invasive liver biopsies that are not feasible in grazing yaks under field conditions—the elevated serum arginine concentrations observed in NCG-supplemented groups are consistent with the activation of this pathway. The elevated arginine, in turn, could activate the mTOR signaling pathway and enhances systemic NO production, which may improve insulin sensitivity and glucose uptake in peripheral tissues. Enhanced glycolysis would be expected to generate pyruvate, which could undergo transamination to produce alanine, offering a potential explanation for the elevated serum alanine levels observed in NCG-supplemented groups. Furthermore, arginine-mediated mTOR activation might promote the expression of amino acid transporters, facilitating the cellular uptake and systemic circulation of multiple amino acids, including BCAA. The observation that NCG1, but not NCG2, significantly elevated circulating BCAA levels could reflect a dose-dependent utilization pattern: at the higher NCG dose (15 g/d), BCAA may have been preferentially sequestered by the mammary gland to support the enhanced milk production observed in the NCG2 group during the 30–60 day period, thereby attenuating their accumulation in the bloodstream. This hypothesis is supported by in vitro studies showing that in mammary epithelial cells, Arg and Phe function as not only substrates but also signaling molecules, modulating lactation efficiency through mTOR signaling pathway under *in vitro* conditions [32,35]. Furthermore, elevated Arg availability to the mammary gland has been shown to enhance milk production and protein synthesis through upregulation of key regulatory genes involved in mammary development and lactogenesis [36]. The NCG1 group demonstrated significantly higher circulating levels of BCAA (leucine, isoleucine, and valine) compared to NCG2, suggesting that 7.5 g/d may be a more favorable dosage among the tested levels for enhancing amino acid availability and metabolic utilization in yaks [37–39]. All BCAA have been reported to enhance milk fat synthesis in vitro. The consistent quadratic patterns for multiple amino acids at day 30 indicate that 7.5 g/d NCG was associated with the highest circulating BCAA levels, whereas the lower concentrations observed at 15 g/d could reflect enhanced mammary extraction and utilization of these amino acids to support the increased milk production during the 30–60 day period, possibly mediated by mTOR-mediated upregulation of amino acid transporters in mammary epithelial cells [32,40]. All BCAA demonstrated the ability to enhance milk fat synthesis *in vitro*. However, the observation that NCG2 had lower circulating BCAA concentrations than the control group at day 30, yet still exhibited numerically higher milk fat content than the control at day 60, could be interpreted as reflecting enhanced mammary extraction and utilization of these amino acids for milk fat synthesis [41], possibly facilitated by arginine-mediated mTOR signaling and upregulation of amino acid transporters in mammary epithelial cells [40]. However, this interpretation remains speculative in the absence of mammary tissue or arteriovenous difference data, and alternative explanations (e.g., differential splanchnic metabolism) cannot be excluded. The transient nature of the amino acid responses—significant only at day 30 but not at day 60—may indicate an adaptive metabolic response to NCG supplementation, wherein the mammary gland’s enhanced capacity for amino acid uptake over time attenuates the systemic accumulation of these metabolites. This pattern is consistent with the sustained milk yield improvement observed across the entire experimental period. The stability of serum biochemical parameters suggests that NCG supplementation does not appear to disrupt systemic metabolic balance under the conditions of this study. Mixed-model analysis confirmed no significant treatment effects or treatment×time interactions for any serum biochemical parameter. Milk acetone concentrations, measured as an indicator of energy balance and subclinical ketosis, also remained unchanged across all treatment groups, suggesting that NCG supplementation did not adversely affect energy metabolism or increase ketogenic risk in grazing yaks under cold-season conditions. Similar observations have been made with other dietary interventions in dairy cattle: for example, supplementation with rumen-protected rubber seed oil did not alter BUN, TP, or glucose concentrations [42]. This finding is particularly significant for grazing yaks, which must maintain metabolic homeostasis while adapting to environmental stressors and nutritional constraints. From a practical standpoint, our results suggest that 7.5 g/d/head NCG supplementation appears to be the more favorable dose among the tested levels for improving lactation performance in grazing yaks. This moderate dose appears sufficient to elicit beneficial metabolic changes without the diminishing returns observed at higher doses. The cost-effectiveness of this supplementation level enhances its potential for adoption in pastoral systems.

From a practical perspective, the results of this study indicate that NCG supplementation at 7.5 g/head/day showed superior effects among the tested doses for improving lactation performance in grazing yaks. This moderate dose effectively induces beneficial metabolic adaptations while maintaining benefits comparable to those of the higher 15 g dose (although at increased levels, milk production is only slightly higher), thus providing outstanding cost-effectiveness for livestock production systems. The combination of proven efficacy and economic feasibility significantly enhances the practical adoption potential of this supplementation strategy.

## CONCLUSION

This study demonstrates that dietary NCG supplementation exerts divergent dose-dependent effects on lactation performance in grazing yaks. Milk yield increased linearly with NCG dosage, with the 15 g/d group producing the highest yield. In contrast, milk fat percentage responded quadratically, peaking at the 7.5 g/d dose, which also showed the strongest effects on circulating branched-chain amino acids. These findings indicate that moderate NCG supplementation (7.5 g/d) optimally improves milk fat synthesis, while higher doses (15 g/d) further stimulate milk volume without additional benefit on fat content. Body weight was not affected by any dose. Orthogonal polynomial contrast analyses confirmed linear trends for milk yield and quadratic trends for milk fat percentage. Although NCG supplementation did not alter serum biochemical parameters, it transiently elevated circulating concentrations of key free amino acids (arginine, alanine, and valine), with more pronounced effects at 7.5 g/d than at 15 g/d, as confirmed by significant quadratic dose-response relationships. The dose-specific responses suggest a divergence in the metabolic pathways activated by NCG, specifically promoting mammary lipogenesis at moderate doses and amino-acid-driven lactogenic signaling at higher doses, although direct mechanistic evidence is required to validate this hypothesis. Further research is warranted to elucidate the underlying molecular mechanisms, evaluate long-term supplementation effects, and assess potential seasonal variations in yak responses.

## Figures and Tables

**Figure 1:**
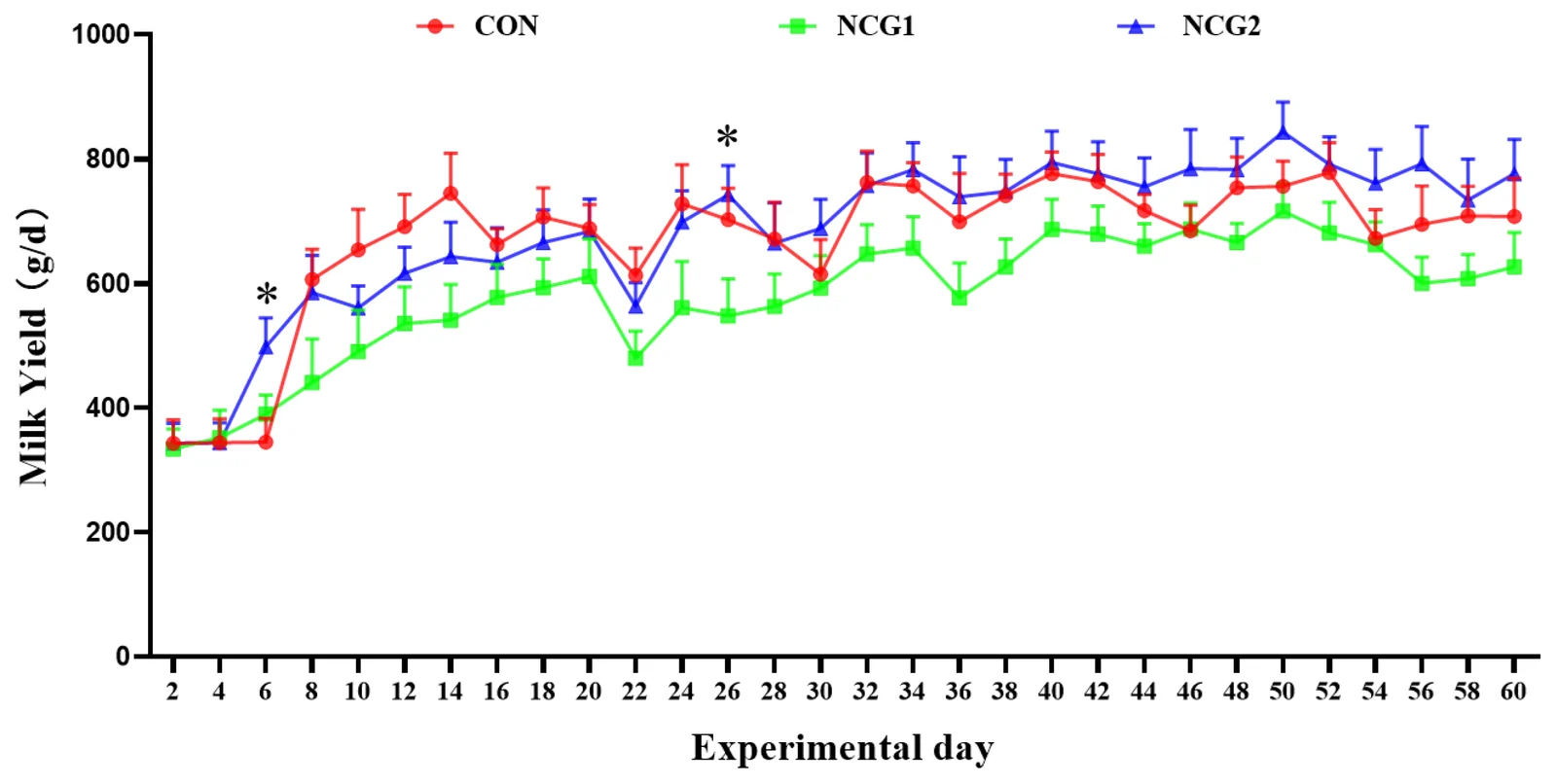
Effects of N-carbamoylglutamate (NCG) supplementation on milk yield in yak cows. Milk production was measured in Control ( 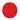) and NCG-supplemented groups (7.5 g/d, 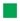; 15 g/d, 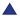). Data were analyzed using linear mixed-effects models with repeated measures, followed by Bonferroni-adjusted pairwise comparisons, and are presented as least squares means±SEM. * indicates significant differences between control and NCG-supplemented groups at the corresponding time point (p<0.05). SEM, standard error of the mean.

**Table 1:** Ingredients and chemical composition of supplementary feed (as fed basis)

Ingredient (%)	
Corn	36.75
Soybean meal	11.00
Calcium carbonate	0.50
Dicalcium phosphate	0.25
Sodium chloride	0.50
Premix1)	1.00
Oat hay	50.00
Total	100.00
Chemical composition (%)2)	
Dry matter	91.53
NEmf (MJ/kg)	5.34
Crude protein	12.07
Ether extract	2.75
Neutral detergent fiber	37.71
Acid detergent fiber	24.12
Calcium	0.41
Phosphorus	0.35

1) The premix provides per kg of diet: 5,500 IU vitamin A, 800 IU vitamin D, 150 IU vitamin E, 150 mg Fe, 60 mg Zn, 50 mg Mn, 10 mg Cu, 0.50 mg I, 0.30 mg Se, and 0.10 mg Co.

2) NEmf was referenced from NY/T815-2004 [23], the others were measured values.

**Table 2:** Effects of NCG on body weight in grazing yaks

Item	Group	Day 0	Day 30	Day 60	SEM	p-value (group)	p-value (time)	p-value (group× time)	Day 0		Day 30		Day 60	

									Linear p-value	Quadratic p-value	Linear p-value	Quadratic p-value	Linear p-value	Quadratic p-value
Body weight (kg)	Control	225.9	217.6	209.0	10.8	0.207	0.130	0.934	0.394	0.215	0.237	0.504	0.154	0.324
	NCG1	252.0	234.7	231.2										
	NCG2	240.6	235	229.3										

Control, yaks received basal supplemental feed at 1% of body weight (BW) without NCG supplementation; NCG1, yaks received basal supplemental feed at 1% BW plus 7.5 g NCG/head/d; NCG2, yaks received basal supplemental feed at 1% BW plus 15 g NCG/head/d.

Data were analyzed using linear mixed-effects models with repeated measures, with treatment group, time, and their interaction as fixed effects, and animal as a random effect.

Body weight was measured on days 0, 30, and 60 of the trial.

Values are presented as least squares means (n = 9 per group).

The SEM value (10.8) was derived from the residual variance of the mixed model and applies to all least squares means presented in this table.

The p-values for linear and quadratic contrasts represent dose-response trends at each time point.

NCG, N-carbamylglutamate; SEM, standard error of the mean.

**Table 3:** Effects of NCG on average daily milk production changes in grazing yaks

Item	Group	Day 0–30	Day 30–60	SEM	p-value (group)	p-value (time)	p-value (group× time)	Day 0–30		Day 30–60	

								Linear p-value	Quadratic p-value	Linear p-value	Quadratic p-value
Average daily gain (kg/d)	Control	−0.28	−0.28	0.22	0.632	0.451	0.546	0.831	0.327	0.580	0.434
	NCG1	−0.58	−0.35								
	NCG2	−0.19	−0.19								
Milk production (g/d)	Control	507.3^b^	647.1^c^	18.4	<0.001	<0.001	0.320	0.018	0.068	<0.001	0.194
	NCG1	607.8^a^	731.5^b^								
	NCG2	595.6^a^	774.6^a^								

Control, yaks received basal supplemental feed at 1% of body weight (BW) without NCG supplementation; NCG1, yaks received basal supplemental feed at 1% BW plus 7.5 g NCG/head/d; NCG2, yaks received basal supplemental feed at 1% BW plus 15 g NCG/head/d.

Data were analyzed using linear mixed-effects models with repeated measures, with treatment group, time, and their interaction as fixed effects, and animal as a random effect, followed by Bonferroni-adjusted pairwise comparisons.

Average daily gain was calculated for the 0–30 d and 30–60 d periods, respectively.

Values represent least squares means for each treatment group (n = 9).

a–c Different lowercase letters within the same column indicate significant differences among treatment groups at the corresponding time point (p<0.05).

The p-values for linear and quadratic contrasts represent dose-response trends at each time point.

NCG, N-carbamylglutamate; SEM, standard error of the mean.

**Table 4:** Effects of NCG on milk composition in grazing yaks

Item	Group	Day 30	Day 60	SEM	p-value (group)	p-value (time)	p-value (group× time)	Day 30		Day 60	

								Linear p-value	Quadratic p-value	Linear p-value	Quadratic p-value
Milk fat (%)	Control	5.70	4.25^b^	0.42	0.172	<0.001	0.969	1.000	0.284	0.726	0.041
	NCG1	6.38	5.12^a^								
	NCG2	5.70	4.40^b^								
Milk protein (%)	Control	5.87	5.55	0.24	0.712	0.122	0.582	0.609	0.957	0.801	0.230
	NCG1	5.95	5.92								
	NCG2	6.06	5.63								
Lactose (%)	Control	5.47	5.50	0.17	0.731	0.638	0.829	0.692	0.707	0.871	0.416
	NCG1	5.58	5.72								
	NCG2	5.56	5.54								
Milk urea nitrogen (mg/dL)	Control	14.2	12.6	1.31	0.760	0.752	0.620	0.897	0.893	0.355	0.574
	NCG1	14.3	14.5								
	NCG2	14.0	14.4								
Acetone (mg/L)	Control	2.50	1.18	0.61	0.359	0.011	0.373	0.334	0.998	0.863	0.039
	NCG1	3.00	2.50								
	NCG2	3.51	1.30								

Control, yaks received basal supplemental feed at 1% of body weight (BW) without NCG supplementation; NCG1, yaks received basal supplemental feed at 1% BW plus 7.5 g NCG/head/d; NCG2, yaks received basal supplemental feed at 1% BW plus 15 g NCG/head/d.

Data were analyzed using linear mixed-effects models with repeated measures, with treatment group, time, and their interaction as fixed effects, and animal as a random effect, followed by Bonferroni-adjusted pairwise comparisons.

Acetone was measured as a biomarker of energy balance and ketosis risk.

Values represent least squares means for each treatment group (n = 9).

a,b Different lowercase letters within the same column indicate significant differences among treatment groups at the corresponding time point (p<0.05).

The p-values for linear and quadratic contrasts represent dose-response trends at each time point.

NCG, N-carbamylglutamate; SEM, standard error of the mean.

**Table 5:** Effects of NCG on serum biochemical parameters in grazing yaks

Item	Group	Day 30	Day 60	SEM	p-value (group)	p-value (time)	p-value (group× time)	Day 30		Day 60	

								Linear p-value	Quadratic p-value	Linear p-value	Quadratic p-value
TP(g/L)	Control	61.5	62.0	0.19	0.693	0.186	0.367	0.155	0.815	0.901	0.427
	NCG1	62.2	60.4								
	NCG2	63.3	61.7								
Albumin (g/L)	Control	29.3	29.6	0.68	0.574	0.692	0.399	0.431	0.454	0.944	0.277
	NCG1	30.5	30.4								
	NCG2	30.2	29.6								
Globulin (g/L)	Control	32.2	32.3	1.49	0.448	0.427	0.671	0.610	0.192	0.933	0.276
	NCG1	30.4	30.0								
	NCG2	33.1	32.1								
Glucose (mmol/L)	Control	3.38	3.78	0.07	0.329	<0.001	0.252	0.058	0.427	0.947	0.620
	NCG1	3.56	3.82								
	NCG2	3.59	3.78								
BUN (mmol/L)	Control	2.63	3.01	0.40	0.080	0.167	0.433	0.106	0.619	0.093	0.154
	NCG1	2.82	2.76								
	NCG2	3.41	4.13								
TC (mmol/L)	Control	3.79	3.71	0.26	0.656	0.101	0.430	0.764	0.640	0.387	0.366
	NCG1	3.89	3.84								
	NCG2	3.67	3.40								
NEFA (μmol/L)	Control	92.2	116.2	6.07	0.311	0.004	0.156	0.603	0.908	0.094	0.608
	NCG1	92.7	111.3								
	NCG2	93.7	95.6								

Control, yaks received basal supplemental feed at 1% of body weight (BW) without NCG supplementation; NCG1, yaks received basal supplemental feed at 1% BW plus 7.5 g NCG/head/d; NCG2, yaks received basal supplemental feed at 1% BW plus 15 g NCG/head/d.

Data were analyzed using linear mixed-effects models with repeated measures, with treatment group, time, and their interaction as fixed effects, and animal as a random effect.

Values represent least squares means for each treatment group (n = 9).

No significant treatment effects or treatment×time interactions were detected for any serum biochemical parameter (p>0.05).

The p-values for linear and quadratic contrasts represent dose-response trends at each time point.

NCG, N-carbamylglutamate; SEM, standard error of the mean; TP, total protein; BUN, blood urea nitrogen; TC, total cholesterol; NEFA, non-esterified fatty acids.

**Table 6:** Effects of NCG on serum free amino acids in grazing yaks

Item	Group	Day 30	Day 60	SEM	p-value (group)	p-value (time)	p-value (group× time)	Day 30		Day 60	

								Linear p-value	Quadratic p-value	Linear p-value	Quadratic p-value
Aspartate (mg/L)	Control	1.44	1.47	0.13	0.046	0.405	0.638	0.207	0.265	0.387	0.044
	NCG1	1.71	1.95								
	NCG2	1.64	1.65								
Glutamate (mg/L)	Control	7.96	7.69	0.66	0.262	0.260	0.887	0.870	0.056	0.586	0.524
	NCG1	8.93	8.03								
	NCG2	7.87	7.03								
Serine (mg/L)	Control	8.11	11.49	0.72	0.204	0.001	0.067	0.940	0.555	0.034	0.319
	NCG1	7.71	9.10								
	NCG2	8.17	8.80								
Glycine (mg/L)	Control	16.7	21.4	1.60	0.678	0.057	0.419	0.824	0.689	0.243	0.823
	NCG1	17.4	19.1								
	NCG2	17.0	18.0								
Histidine (mg/L)	Control	5.85	6.07	0.38	0.056	0.207	0.395	0.340	0.610	0.028	0.500
	NCG1	5.84	5.02								
	NCG2	5.37^b^	4.67								
Arginine (mg/L)	Control	17.5^a^	18.2	0.97	0.007	0.200	0.929	0.141	0.045	0.124	0.133
	NCG1	20.5^ab^	21.7								
	NCG2	19.2	20.7								
Threonine (mg/L)	Control	1.68	2.19	0.27	0.460	0.531	0.441	0.402	0.233	0.347	0.533
	NCG1	2.15	2.22								
	NCG2	1.95	1.78								
Alanine (mg/L)	Control	9.32^b^	15.55	2.28	0.083	0.006	0.328	0.068	0.018	0.711	0.077
	NCG1	14.4^a^	21.7								
	NCG2	12.4^ab^	13.9								
Proline (mg/L)	Control	12.2	15.1	1.35	0.356	0.036	0.541	0.520	0.260	0.565	0.241
	NCG1	13.7	16.9								
	NCG2	12.9	13.6								
Tyrosine (mg/L)	Control	4.13	4.20	0.53	0.241	0.532	0.909	0.328	0.208	0.286	0.457
	NCG1	5.10	5.27								
	NCG2	4.73	5.17								
Valine (mg/L)	Control	15.9^b^	15.1	1.41	0.090	0.334	0.982	0.545	0.010	0.655	0.241
	NCG1	18.5^a^	17.2								
	NCG2	15.2^b^	14.0								
Methionine (mg/L)	Control	0.79	0.97	0.13	0.251	0.402	0.544	0.400	0.165	0.609	0.190
	NCG1	1.04	1.16								
	NCG2	0.92	0.86								
Isoleucine (mg/L)	Control	10.00^ab^	7.28	0.79	0.081	0.014	0.576	0.054	0.076	0.989	0.224
	NCG1	10.50^a^	8.75								
	NCG2	8.28^b^	7.29								
Leucine (mg/L)	Control	8.21^ab^	7.42	0.74	0.046	0.291	0.986	0.249	0.015	0.671	0.184
	NCG1	9.40^a^	8.71								
	NCG2	7.39^b^	6.86								
Phenylalanine (mg/L)	Control	4.07^b^	4.26	0.40	0.157	0.450	0.558	0.021	0.038	0.992	0.346
	NCG1	5.14^a^	4.87								
	NCG2	4.91^a^	4.27								
Lysine (mg/L)	Control	5.25	5.54	0.54	0.189	0.706	0.907	0.866	0.087	0.618	0.220
	NCG1	6.06	6.33								
	NCG2	5.16	5.06								

Control, yaks received basal supplemental feed at 1% of body weight (BW) without NCG supplementation; NCG1, yaks received basal supplemental feed at 1% BW plus 7.5 g NCG/head/d; NCG2, yaks received basal supplemental feed at 1% BW plus 15 g NCG/head/d.

Data were analyzed using linear mixed-effects models with repeated measures, with treatment group, time, and their interaction as fixed effects, and animal as a random effect, followed by Bonferroni-adjusted pairwise comparisons.

Values represent least squares means for each treatment group (n = 9).

a,b Different lowercase letters within the same column indicate significant differences among treatment groups at the corresponding time point (p<0.05).

The p-values for linear and quadratic contrasts represent dose-response trends at each time point.

NCG, N-carbamylglutamate; SEM, standard error of the mean.

## Data Availability

Upon reasonable request, the datasets of this study can be available from the corresponding author.
